# Increased Brown Adipose Tissue Oxidative Capacity in Cold-Acclimated Humans

**DOI:** 10.1210/jc.2013-3901

**Published:** 2014-01-13

**Authors:** Denis P. Blondin, Sébastien M. Labbé, Hans C. Tingelstad, Christophe Noll, Margaret Kunach, Serge Phoenix, Brigitte Guérin, Éric E. Turcotte, André C. Carpentier, Denis Richard, François Haman

**Affiliations:** Faculty of Health Sciences (D.P.B., H.C.T., F.H.), University of Ottawa, Ottawa, Ontario, Canada K1N 6N5; Centre de Recherche de l'Institut Universitaire de Cardiologie et de Pneumologie de Québec (S.M.L., D.R.), Université Laval, Québec City, Québec, Canada G1V 4G5; Department of Medicine (C.N., M.K., S.P., A.C.C.), Centre de Recherche Clinique Etienne-Le Bel, Université de Sherbrooke, Sherbrooke, Québec, Canada; and Department of Nuclear Medicine and Radiobiology (S.P., B.G., E.E.T.), Université de Sherbrooke, Sherbrooke, Québec, Canada J1H 5N4

## Abstract

**Context:**

Recent studies examining brown adipose tissue (BAT) metabolism in adult humans have provided convincing evidence of its thermogenic potential and role in clearing circulating glucose and fatty acids under acute mild cold exposure. In contrast, early indications suggest that BAT metabolism is defective in obesity and type 2 diabetes, which may have important pathological and therapeutic implications. Although many mammalian models have demonstrated the phenotypic flexibility of this tissue through chronic cold exposure, little is known about the metabolic plasticity of BAT in humans.

**Objective:**

Our objective was to determine whether 4 weeks of daily cold exposure could increase both the volume of metabolically active BAT and its oxidative capacity.

**Design:**

Six nonacclimated men were exposed to 10°C for 2 hours daily for 4 weeks (5 d/wk), using a liquid-conditioned suit. Using electromyography combined with positron emission tomography with [^11^C]acetate and [^18^F]fluorodeoxyglucose, shivering intensity and BAT oxidative metabolism, glucose uptake, and volume before and after 4 weeks of cold acclimation were examined under controlled acute cold-exposure conditions.

**Results:**

The 4-week acclimation protocol elicited a 45% increase in BAT volume of activity (from 66 ± 30 to 95 ± 28 mL, *P* < .05) and a 2.2-fold increase in cold-induced total BAT oxidative metabolism (from 0.725 ± 0.300 to 1.591 ± 0.326 mL·s^−1^, *P* < .05). Shivering intensity was not significantly different before compared with after acclimation (2.1% ± 0.7% vs 2.0% ± 0.5% maximal voluntary contraction, respectively). Fractional glucose uptake in BAT increased after acclimation (from 0.035 ± 0.014 to 0.048 ± 0.012 min^−1^), and net glucose uptake also trended toward an increase (from 163 ± 60 to 209 ± 50 nmol·g^−1^·min^−1^).

**Conclusions:**

These findings demonstrate that daily cold exposure not only increases the volume of metabolically active BAT but also increases its oxidative capacity and thus its contribution to cold-induced thermogenesis.

In the 4 years since the seminal papers describing the presence of functional brown adipose tissue (BAT) in adult humans were published (1–3), significant progress has been made in characterizing its developmental origin (4, 5), function (6, 7), and distribution (8, 9). As in most mammals, BAT provides an important contribution to thermoregulatory heat production in adult humans (6). The scarce amount of BAT found in individuals who are overweight or obese (10, 11) combined with the large BAT mass and lean phenotype exhibited by patients with pheochromocytoma (catecholamine-secreting tumors) (12, 13) have led to suggestions that it may also play an important part in energy homeostasis. Whether BAT can be induced to grow or increase its metabolic capacity and consequently be a therapeutic target for obesity and its related complications remains unclear. Evidence from animal models (14) and indirect support from human studies (15, 16) have suggested an inherent plasticity to this tissue, presenting signs of BAT recruitment. Furthermore, the differentiation of both types of brown adipocytes (classical and beige/brite) (4, 17, 18) and its activation (1, 3, 6) appear to be largely mediated by a sympathetic input. To date, the most potent and effective sympathetic stimulator of BAT differentiation and activation in humans has been cold exposure, as most sympathomimetics applied in vivo have shown little to no effect on BAT activation (19–21). Very recent studies have demonstrated that relative BAT glucose uptake as assessed by increased [^18^F]fluorodeoxyglucose (^18^FDG) standard uptake value (SUV) with positron emission tomography (PET) can be increased by repeated cold exposure in humans (22, 23). However, whether BAT oxidative capacity increases with the increase in BAT recruitment has not yet been demonstrated in adult humans. Six healthy lean men were invited to participate in a 4-week cold-acclimation protocol and undergo acute cold-exposure studies before and after the intervention to determine whether 4 weeks of daily cold exposure could increase both the volume of metabolically active BAT and its oxidative capacity.

## Materials and Methods

### Experimental protocol

**Figure 1.**
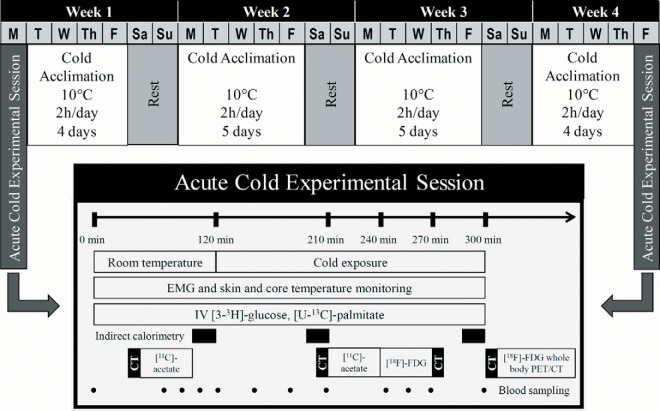
Study protocol.

Six healthy lean men aged 23 ± 1 year with a body mass index of 24.5 ± 1.2 kg/m^2^ and body surface area of 2.01 ± 0.04 m^2^ participated in 2 metabolic experimental sessions within a 4-week interval designed to assess whole-body and tissue-specific metabolism during an acute cold exposure (Figure 1). The cold acclimation protocol followed between these 2 experimental sessions consisted of daily cold exposure lasting 2 hours during which 10°C water was circulated through a liquid-conditioned suit (Three Piece; Allen-Vanguard), repeated 5 consecutive days per week for 4 consecutive weeks for a total of 18 acclimation sessions and 2 testing sessions. Participants were asked to maintain their current training regiment and refrain from drinking caffeinated or alcoholic beverages for the duration of the study.

Each acute cold exposure experimental session consisted of a 120-minute baseline period at ambient temperature (∼25°C) followed by 180 minutes of exposure to a mild cold, elicited using the same liquid-conditioned suit. During cold exposure, 18.0°C water was circulated through the suit during the preacclimation visit and water at a temperature eliciting the same individualized inlet-outlet temperature difference as the preacclimation protocol (16.8°C ± 0.1°C) was circulated during the postacclimation visit using a temperature-controlled circulation bath (Endocal, NESLAB Model 200–00; Micropump). The postacclimation temperature was adjusted to induce a similar thermogenic rate between the 2 experimental sessions. The same suit was used for all subjects to maintain consistent tubing density and water flow. Experiments were conducted between 7:30 am and 4:00 pm hours, after 48 hours without strenuous physical activity. Upon their arrival in the laboratory, subjects wearing only shorts were weighed and instrumented with 12 autonomous wireless temperature sensors (Thermochron iButton model DS1922H, Maxim) fixed to the skin to measure mean skin temperature (24) and surface electromyography (EMG) electrodes (Delsys; EMG Systems) placed on the belly of 12 muscles. Participants were then fitted with the liquid-conditioned suit, ingested a telemetric thermometry capsule to measure core temperature (Vital Sense monitor and Jonah temperature capsule; Mini Mitter Co, Inc), and performed a series of exercises to estimate the maximal voluntary contraction of each of the muscles being measured for shivering activity. Whole-body metabolic heat production was determined by indirect respiratory calorimetry (Vmax 29n; Sensormedics) (25) at room temperature and between 180 to 200 minutes and 280 to 300 minutes (ie, 60 to 80 minutes and 160 to 180 minutes after the beginning of cold exposure). Whole-body and muscle-specific shivering intensity and pattern as well as mean skin and core temperatures were measured continuously from time 90 to 300 minutes as previously described (26). Only the means of the final 30 minutes of the ambient period and final 120 minutes of the cold exposure are reported. A weighted average of the ^18^FDG uptake of 18 skeletal muscles, which includes deep muscles that are inaccessible using surface EMG, was also used as a shivering index to determine whether 1) shivering activity was modified based on muscle location (superficial vs deep) and 2) ^18^FDG uptake in skeletal muscles could serve as a viable indicator of whole-body shivering activity. Plasma glucose appearance rate was determined using a primed continuous infusion (0.33 × 106 dpm/min) of [3-^3^H]glucose (27). The rate of appearance of nonesterified fatty acids (NEFAs) was measured using iv administration of [U-^13^C]palmitate using the Steele steady-state equation, as previously described (28).

### PET/computed tomography protocol

Tissue oxidative metabolism was determined by first performing a computed tomography (CT) scan (40 milliAmpere-second) centered at the cervicothoracic junction to correct for attenuation and to define PET regions of interest. At 90 minutes (room temperature) and again at 210 minutes (ie, 90 minutes after onset of cold exposure), ∼185 MBq of [^11^C]acetate was injected iv followed by a 30-minute list-mode dynamic PET acquisition (24 × 10 seconds, 12 × 30 seconds, 4 × 300 seconds), as previously described (29). The tissue oxidative metabolism index (the rapid fractional tissue clearance of ^11^C-acetate, *k*, in s^−1^) was estimated from tissue ^11^C activity over time using monoexponential fit from the time of peak tissue activity (30). This method is based on the following assumptions (31): 1) acetate enters the Krebs cycle freely after rapid conversion into acetyl-coenzyme A; 2) other acetate metabolic fates (eg, de novo lipogenesis) are relatively slow compared with the Krebs cycle carbon fluxes; 3) carbon fluxes into the Krebs cycle through acetyl-coenzyme A are directly coupled to the production of reducing equivalents; 4) the Krebs cycle contribution to the production of reducing equivalents is stable and accounts for approximately two-thirds of total production; and 5) the production of reducing equivalents is tightly coupled to oxygen consumption. Total oxidative metabolism index of BAT was determined by multiplying *k* (representing the oxidative metabolism of a particular depot) by the total volume of metabolically active BAT, as determined by ^18^FDG uptake (described below). This calculation was performed to reflect the total oxidative metabolism of BAT located throughout the body.

To determine tissue glucose uptake, an iv bolus of ^18^FDG (∼185 MBq) was given at 240 minutes (ie, 2 hours after the onset of cold exposure), with a 40-minute list-mode dynamic PET acquisition (12 × 10 seconds, 8 × 30 seconds, 6 × 90 seconds, 5 × 300 seconds), followed by a CT scan (40 mAs) centered at the cervicothoracic junction to correct for attenuation and for definition of PET regions of interest. Plasma and tissue time-radioactivity curves were analyzed graphically using the Patlak linearization method (32), with the image-derived arterial input function taken from the aortic arch (33). The slope of the plot in the graphical analysis is equal to the tissue glucose extraction constant of ^18^FDG (*K*_i_ in min^−1^ of ^18^FDG). Tissue net glucose uptake (*K*_m_) was then calculated by multiplying *K*_i_ by plasma glucose concentration, measured during the PET imaging protocol, which assumes a lump constant value of 1.0 compared with endogenous plasma glucose. After cold exposure (at 300 minutes), a whole-body CT scan (16 mAs) followed by a static whole-body PET acquisition was performed to determine whole body ^18^FDG organ distribution and tissue SUV.

### PET/CT image analyses

The regions of interest were first defined from the transaxial CT slices and then copied to ^18^FDG and then to [^11^C]acetate PET image sequences. For dynamic PET acquisitions, the mean value of pixels (mean SUV) for each frame was recorded. Regions of interest were drawn on the aortic arch for blood activity (input functions), the larger skeletal muscles in the field of view, posterior cervical sc adipose tissue, and the supraclavicular BAT according to the following criteria: a tissue radio density between −30 and −150 Hounsfield units and ^18^FDG uptake during cold exposure of more than 1.5 SUV unit. The total BAT volume of activity on whole-body scans was also quantified according to the latter criteria. For whole-body scans, mean values of pixels (mean SUV) for all tissues of interest were recorded.

### Statistical analysis

Data are expressed as mean ± SEM. Paired Student's *t* test was used to compare between acute cold exposure experimental sessions. Two-way ANOVA for repeated measures with acclimation status, temperature, and their interaction as the independent variables was used to analyze acclimation- and temperature-dependent differences in averaged steady-state hormone and metabolite levels and blood and tissue PET-acquired activities throughout the protocols. Bonferroni's multiple-comparisons post hoc test was used, where applicable. Appropriate transformations of variables were performed when a normal distribution was not observed for parametric statistical testing. Pearson correlation coefficients were used to determine correlation between variables. A 2-tailed *P* value < .05 was considered significant. All analyses were performed using SPSS for Windows version 16.0 or GraphPad Prism version 6.00 for Windows.

### Study approval

Participants were fully informed of the risks and methodologies applied and provided their written consent to participate in this study, in accordance with the Declaration of Helsinki. This study received ethics approval from the Office of Research Ethics and Integrity at the University of Ottawa and the Institutional Review Board for research on humans of the Centre Hospitalier Universitaire de Sherbrooke and Université de Sherbrooke.

## Results

### Effect of cold acclimation on thermal responses and plasma metabolites

**Figure 2.**
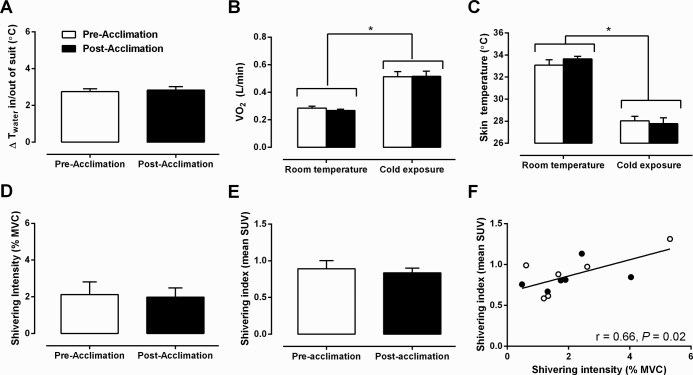
Thermal responses. A, Change in inlet and outlet water temperature of liquid-conditioned garment. B and C, Oxygen consumption (VO_2_) (B) and mean skin temperature (C) during room temperature and cold exposure, before and after acclimation. D and E, Shivering intensity (D) and shivering intensity index (E) before and after acclimation. F, Relationship between mean shivering intensity and shivering intensity index (Pearson *r* = 0.66, *P* = .02). *, *P* < .05 vs room temperature, ANOVA with Bonferroni's post hoc test.

The energy expenditure was individually matched between experimental sessions by maintaining the same difference in inlet and outlet temperature of the water circulating through the cooling garment (Δ2.8°C ± 0.2°C; Figure 2A) before and after the 4-week cold acclimation. This elicited a similar 1.9-fold increase in thermogenic rate (Figure 2B) in the acute cold experimental sessions. Using this approach, the cold stimulus produced by the liquid-conditioned cooling garment evoked a decrease in mean skin temperature that was the same between acute cold-exposure sessions (Figure 2C). Shivering intensity, which was purposely kept to a minimum, was not significantly different between experimental conditions, whether it was determined electromyographically (Figure 2D) or using a weighted average of the ^18^FDG uptake of 18 skeletal muscles as a shivering index (Figure 2E). The significant relationship between shivering intensity and the shivering index (Pearson *r* = 0.66, *P* = .02) suggests that the latter may also represent a good indicator of whole-body shivering activity, which includes deep muscles that are inaccessible using surface EMG (Figure 2F).

To assess the whole-body metabolic consequences of daily cold exposure, hormonal and metabolite changes were examined. Insulin, triglyceride (TG), T_3_, T_4_, ACTH, and leptin levels did not change significantly with cold exposure or cold acclimation (Table 1). NEFA rate of appearance, oxidation rate, and concentration were similarly increased before and after cold acclimation during acute cold exposure. Only glucose and cortisol concentrations appeared to be influenced by acclimation state, with both being significantly lower after compared with before acclimation, regardless of temperature exposure. Glucose production rate was not significantly changed after cold acclimation, demonstrating that the reduced glucose level was caused by increased glucose clearance.

**Table 1.** Hormone and Metabolite Concentrations at Room Temperature and Cold Exposure, Before and After Cold Acclimation

	Before Acclimation		After Acclimation	
	Room Temperature	Cold Exposure	Room Temperature	Cold Exposure
Energy expenditure, kcal/min	1.4 ± 0.1	2.7 ± 0.2^b^	1.3 ± 0.0	2.5 ± 0.2^b^
Glucose, mmol/L	4.8 ± 0.2	4.8 ± 0.1	4.6 ± 0.1^c^	4.5 ± 0.1^c^
Ra_glucose_, μmol/min		1707 ± 166		2059 ± 100
Insulin, pmol/L	67 ± 13	53 ± 8	57 ± 14	53 ± 9
TG, mmol/L	1.20 ± 0.47	1.13 ± 0.41	0.95 ± 0.36	1.02 ± 0.41
NEFA, μmol/L	398 ± 53	687 ± 110^b^	411 ± 69	691 ± 132^b^
Ra_NEFA_, μmol/min	485 ± 71	756 ± 96^b^	448 ± 111	665 ± 119^b^
Rox_NEFA_, μmol/min	326 ± 77	601 ± 89^b^	319 ± 65	631 ± 128^b^
TSH, IU/L	2.56 ± 0.73	1.96 ± 0.54^b^	1.98 ± 0.34	1.82 ± 0.47^b^
Free T_3_, pmol/L	6.0 ± 0.4	5.8 ± 0.4	5.8 ± 0.3	7.0 ± 1.1
Free T_4_, pmol/L	16.2 ± 0.8	16.7 ± 0.7	16.8 ± 0.4	16.0 ± 1.5
ACTH, pmol/L	4.2 ± 0.7	3.4 ± 0.4	3.8 ± 0.2	3.4 ± 0.4
Cortisol, nmol/L	374 ± 25	308 ± 37	299 ± 48^c^	280 ± 27^c^
Leptin, ng/mL	2.5 ± 0.9	2.4 ± 1.0	2.6 ± 0.9	2.3 ± 0.7

Abbreviations: Ra, rate of appearance; Rox, oxidation rate.

a Values are means ± SEM; n = 6 subjects.

b Different from room temperature, *P* < .05.

c Different from Pre-acclimation, *P* < .05.

### Daily cold exposure increases BAT volume of activity and fractional glucose uptake

**Figure 3.**
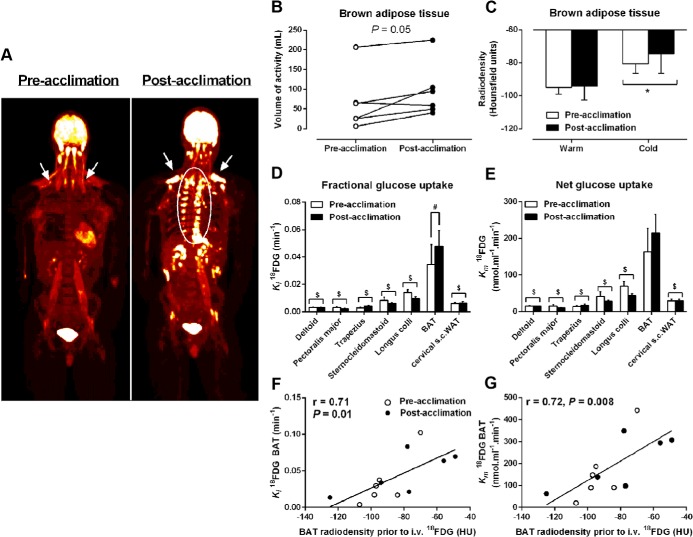
Tissue glucose uptake. A, Coronal view (anterior-posterior projection) of whole-body ^18^FDG uptake during cold exposure before and after acclimation. B, Volume of BAT ^18^FDG activity before and after acclimation. C, BAT radiodensity by CT during room temperature and cold exposure before and after acclimation. D and E, Fractional (*K*_i_) (D) and net (*K*_m_) (E) glucose uptake in cervicothoracic tissues. F and G, Relationship between BAT radiodensity from CT taken before iv ^18^FDG injection in the cold and *K*_i_^18^FDG (Pearson r = 0.71, *P* = .01) (F) and *K*_m_^18^FDG (Pearson *r* = 0.72, *P* = .008) (G). *, *P* < .05 vs room temperature; $, *P* < .005 vs BAT; #, *P* < .05 vs before acclimation, ANOVA with Bonferroni's post hoc test.

To examine the effect of daily cold exposure on BAT volume, we determined the whole-body volume of ^18^FDG uptake in BAT (ie, volume of BAT activity) after a whole-body PET/CT acquisition performed immediately upon completing the acute cold exposure. A whole-body PET/CT image of a representative participant before and after a 4-week cold acclimation protocol is shown in Figure 3A. Total BAT volume of activity increased by 45% after 4 weeks of cold acclimation (66 ± 30 mL before acclimation vs 95 ± 28 mL after acclimation; *P* = .05; Figure 3B). BAT attenuation, determined using CT and expressed in Hounsfield units, was the same at room temperature and increased to a similar degree in all participants after an acute cold exposure, independent of acclimation status (Figure 3C). To examine cold-stimulated BAT and skeletal muscle quantitative glucose uptake, a cervicothoracic dynamic PET/CT acquisition was performed after the iv injection of a bolus of ^18^FDG during the acute cold exposure. Fractional uptake (*K*_i_) of ^18^FDG (Figure 3D) was significantly greater in supraclavicular BAT compared with the longus colli, sternocleidomastoid, trapezius, pectoralis major, and deltoid muscles as well as sc adipose tissue. The *K*_i_ of ^18^FDG in supraclavicular BAT was significantly greater after acclimation compared with before acclimation to cold (Figure 3D). Similarly, net tissue glucose uptake (*K*_m_) (Figure 3E) was significantly higher in BAT vs longus colli, sternocleidomastoid, trapezius, pectoralis major, and deltoid muscles as well as sc adipose tissue. The *K_m_* of ^18^FDG in supraclavicular BAT was not significantly different between acclimation states (*P* = .08). Given a total glucose uptake by BAT of 20.1 ± 15.2 μmol/min before acclimation and 26.2 ± 11.8 μmol/min after acclimation (*P* = .08) and a plasma glucose appearance of 1707 ± 166 and 2059 ± 100 μmol/min, respectively, BAT glucose uptake accounted for 1.1% ± 0.8% and 1.4% ± 0.7% of plasma glucose turnover, respectively. There was a significant direct relationship between the radiodensity of BAT and the fractional and net glucose uptake by the tissue (Pearson *r* = 0.71, *P* = .01; and Pearson *r* = 0.72, *P* = .008, respectively; Figure 3, F and G).

### Daily cold exposure increases BAT oxidative metabolism

**Figure 4.**
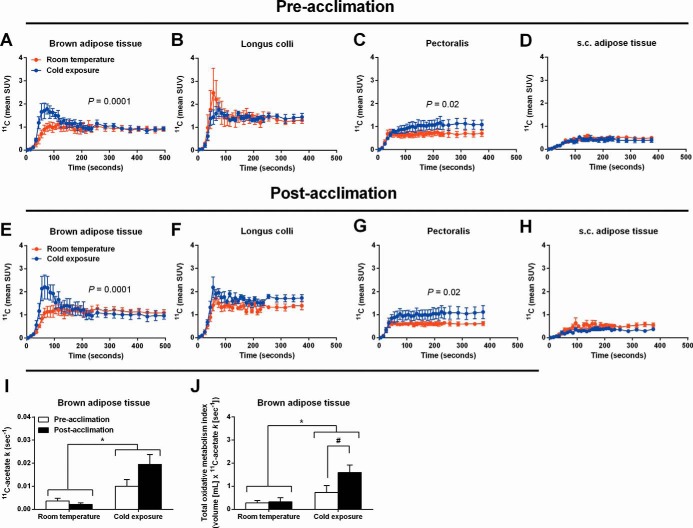
[^11^C]Acetate kinetics. A–H, ^11^C time-radioactivity curves over the first 500 seconds of acquisition after [^11^C]acetate injection at room temperature (red) and during cold exposure (blue) in BAT (A and E), longus colli (B and F), pectoralis (C and G), and cervical sc white adipose tissue (D and H) before and after acclimation. I, Tissue oxidative metabolism index ([^11^C]acetate *k*) in cervicothoracic BAT before and after acclimation. J, Total BAT oxidative metabolism index before and after acclimation. *, *P* < .05 vs room temperature; #, *P* < .05 vs before acclimation, ANOVA with Bonferroni's post hoc test.

To investigate the oxidative capacity of cold-stimulated BAT, a cervicothoracic dynamic PET/CT acquisition was performed after the iv injection of a bolus of [^11^C]acetate during the acute cold exposure. After the iv injection of [^11^C]acetate, BAT ^11^C radioactivity over time was significantly higher during cold exposure compared with room temperature, both before and after a cold acclimation intervention (Figure 4, A and E). Pectoralis major was the only muscle displaying a significant cold-induced increase in ^11^C radioactivity over time (Figure 4, C and G). The monoexponential decay slope from tissue peak ^11^C activity ([^11^C]acetate *k*), a surrogate of tissue oxidative metabolism (34, 35), increased significantly during cold exposure in BAT (effect of temperature *P* = .001; Figure 4I), demonstrating an increase in cold-induced oxidative metabolism. The monoexponential decay slope from tissue peak ^11^C activity was also presented as a function of the total BAT volume of activity to demonstrate the total oxidative metabolism of BAT. The total BAT oxidative metabolism increased significantly in the cold with the increase being significantly greater after the cold acclimation (temperature × acclimation interaction *P* = .02; Figure 4J).

## Discussion

Based on the PET tracers ^18^FDG and [^11^C]acetate, this study demonstrates for the first time that daily cold exposure not only increases the volume of metabolically active BAT by 45% but also doubles its cold-induced oxidative capacity in adult humans. Within the [^11^C]acetate PET acquisition field of view including the neck and upper thorax, BAT was the only tissue demonstrating a significant increase in the total oxidative metabolism in the cold with the increase being significantly greater after the cold acclimation. [^11^C]Acetate as PET tracer has proved to be instrumental in assessing BAT (6) and other tissue metabolism in vivo (31, 32, 36, 37).

Thirty years ago, Huttunen et al (16) described the greater presence of multilocular adipocytes after necropsy in adipose tissue samples excised from the neck region of Finnish outdoor workers exposed to the cold compared with indoor workers. Until recently, this was the only evidence suggesting that chronic cold exposure induces increases in BAT mass in adult humans. However, the hypothesis that chronic cold activation stimulates BAT recruitment in vivo in adult humans has only recently explicitly been investigated (22, 23), while the present study was in progress. We and others (22) have observed that daily exposure to a mild cold, an unequivocal and to date most potent and safe stimulus to activate BAT in humans, does indeed increase whole-body BAT volume of activity, as defined by the volume of ^18^FDG uptake in BAT. Recent studies have demonstrated that most BAT depots in humans exhibit molecular signatures and histological features resembling the inducible brown adipocytes (known as beige, brown-in-white, or brite) clustered within white adipose tissue depots of cold-exposed rodents (4, 5) but may also contain classical BAT (9, 38). Whether the increased volume of glucose uptake observed herein is a result of proliferation of classical brown adipocytes, a de novo recruitment of brite adipocytes from white adipose tissue precursors (4), or simply a direct interconversion of mature white adipocytes into a brown adipocyte phenotype or browning (39, 40), is unclear. These findings imply that the thermogenic potential of BAT has increased as a result of the 4-week cold acclimation. Due to the requirement of using radioactive isotopes to assess BAT metabolism in vivo in humans and limits in radioactivity exposure, the time course of both BAT recruitment and atrophy after the end of the intervention are not possible to determine in a within-subject design such as this.

The decreases in BAT radiodensity and the low levels of glucose uptake, averaging 20.1 ± 15.2 and 26.2 ± 11.8 μmol/min, under both experimental cold conditions lends further support to the notion that intracellular TGs are likely the predominant substrate fueling BAT oxidative metabolism in humans. Although intracellular TG depots decrease during the acute cold exposure, the similar radiodensity of BAT observed under unstimulated conditions (eg, room temperature) before and after acclimation would suggest that they replete rapidly after the acute cold exposure. The time course of this repletion, including the role of BAT in postprandial metabolism, requires further investigation and may shed some light on the metabolic regulatory function of BAT. The significant inverse relationship between BAT radiodensity and its fractional and net glucose uptake suggests that, similar to skeletal muscle, circulating substrates may supply and complement the intracellular depots to meet the energy demand under stimulated conditions. The metabolic fate of glucose taken up by BAT has yet to be clearly established in humans. However, with BAT thermogenesis dependent on fatty acids for the activation of uncoupling protein-1 (UCP1) and as a substrate to fuel thermogenesis (14), glucose is likely supplying the carbon backbone for fatty acid synthesis, which can be subsequently oxidized. The reduced concentration of circulating glucose after acclimation, despite a greater glucose rate of appearance, combined with a trend toward a greater glucose clearance by BAT also suggests that this tissue may play a greater role in glucose metabolism than previously suspected. The detailed characterization of the fuel utilization and substrate handling of this tissue in humans warrants further investigation if it is to be further pursued as a therapeutic target for metabolic diseases.

The metabolic impact of the increased BAT oxidative capacity that occurred after repetitive cold exposures may have contributed to an increase in the nonshivering thermogenesis (NST) of our subjects. Nonetheless, our cold acclimation protocol was insufficient to promote the recruitment of BAT-mediated NST to the extent necessary to completely abolish the shivering response (Figure 2). More prolonged and/or intense cold acclimation could be necessary to fully recruit BAT-mediated NST in humans, or exposure to a colder acute thermal challenge may be required for BAT-mediated NST to fully manifest. Furthermore, although EMG was monitored in 12 muscle groups, it remains that this method measures superficial EMG activity and provides some insight on fiber recruitment and fuel selection. Consequently, it is possible that deeper muscle groups, not accessible by EMG, were most influenced by the changes in BAT-mediated NST or that changes in skeletal muscle bioenergetics were also modified. In the only cold acclimation study quantifying shivering activity in humans, and thus de facto NST, 20 days of daily exposure to 12°C (8 h/d) was required to observe a near abolishment of shivering activity in men previously acclimated to summer conditions (41). It is noteworthy that even in rodents subjected to aggressive cold-exposure protocols (often 24 hours exposure for several days at 4°C), NST develops progressively (42). In rats, cold exposure leads to an initial (a few hours) increase in BAT thermogenic activity associated with an increase in UCP1 stimulation, which is followed after a few days by a progressive increase in the thermogenic capacity, revealed through increases in UCP1 expression, mitochondriogenesis, and brown adipocyte protein content (43). In rats, cold adaptation has been reported to tremendously increase the thermogenic capacity of BAT, which can account for more than 60% of the total heat produced in response to noradrenaline with little contribution from skeletal muscle (44). In the present study, because we did not have access to BAT samples, we cannot determine the cause of the increase in BAT thermogenic capacity (ie, increase in expression of UCP1 and accessory thermogenic genes or mitochondriogenesis). Another important limitation of the present study is the inability to assess the relative contribution of BAT and muscle as well as organs such as the heart and liver to total thermogenesis. Nevertheless, based on the present [^11^C]acetate oxidative metabolism and BAT radiodensity data, one can be confident of some contribution of BAT to energy metabolism after 4 weeks of acclimation, which is in line with evidence showing that human brown adipocytes are metabolically active and share similarities with classic brown adipocytes seen in laboratory rodents (9).

In summary, we showed that total BAT volume of activity increases significantly as a result of repeated controlled daily cold exposure and that this change in mass is paralleled by an increase in BAT oxidative capacity. Therefore, the contribution of BAT to NST must necessarily increase during cold acclimation in humans.
